# Sexual behaviours and lifestyles associated with poor mental health among British adults during the early phase of the COVID-19 pandemic: findings from a large, quasirepresentative, cross-sectional web-panel survey (Natsal-COVID)

**DOI:** 10.1136/bmjph-2024-001443

**Published:** 2025-05-12

**Authors:** Emily Jordan, Emily Dema, Soazig Clifton, Andrew Copas, Julie Riddell, Raquel Bosó Pérez, Catherine H Mercer, Kirstin Rebecca Mitchell, Nigel Field, Pam Sonnenberg

**Affiliations:** 1 University College London, London, UK; 2 University of Glasgow, Glasgow, UK

**Keywords:** COVID-19, Mental Health, Sex Factors, Population Surveillance

## Abstract

**Objective:**

A national lockdown was implemented in the UK from March 2020 to reduce COVID-19 transmission which impacted mental health and sexual behaviours. This study investigated the association between sexual behaviours and poor mental health among British adults during the early phase of the COVID-19 pandemic.

**Methods and analysis:**

The Natsal-COVID web-panel survey is a quasirepresentative population sample of 6654 British adults (18–59 years; July–August 2020). We examined associations between sexual behaviours in the 4 months after the start of the first lockdown and poor mental health in the past 2 weeks (Patient Health Questionnaire (PHQ)-2 for depression and Generalised Anxiety Disorder-2 for anxiety, combined into PHQ-4 for psychological distress) using multivariable logistic regression.

**Results:**

Among participants with complete data (n=6500), 54.8% reported psychological distress (28.0% mild, 15.6% moderate and 11.3% severe). After adjusting for age, gender and relationship status, psychological distress was higher among participants identifying as bisexual (adjusted OR 2.13 (95% CI: 1.61 to 2.82)) or other minority sexual identities (3.20, 1.60–6.41) compared with heterosexual. Psychological distress was also higher among those reporting experiencing sexual difficulties (very often/always vs never: 3.70, 2.64–5.18) or 2+partners since lockdown (compared with one: 3.90, 2.78–5.47). Psychological distress was less among participants reporting higher sexual frequency (5+occasions of sex in the past 4 weeks vs none: 0.64, 0.48–0.86).

**Conclusions:**

Psychological distress was common among British adults during the COVID-19 pandemic, and this was associated with sexual behaviours. These findings have important implications for the design and implementation of interventions for populations at risk of poor mental and sexual health, including during international crises.

**WHAT IS ALREADY KNOWN ON THIS TOPIC**

- Population studies across the world have shown that mental illness and psychological distress increased during the initial stages of the COVID-19 pandemic. Access to mental health services was also reduced, and resources diverted from mental healthcare to other areas. Social COVID-19 restrictions had a variety of impacts on sexual behaviours and lifestyles. Cohabiting partners likely spent more time together, while those living separately were prohibited from intimate contact. The inclination to have sex may also have been altered by the stresses associated with the pandemic. Sexual and mental health have been linked, but the relationship has not been widely studied.

**WHAT THIS STUDY ADDS**

- During the COVID-19 pandemic, psychological distress was common among British adults and it was associated with sexual behaviours. After adjusting for age, gender and relationship status, psychological distress was higher among participants identifying as bisexual or other minority sexual identities compared with heterosexuals, reporting experiencing sexual difficulties and reporting two or more partners since lockdown compared with one. Psychological distress was less among participants reporting higher sexual frequency.

**HOW THIS STUDY MIGHT AFFECT RESEARCH, PRACTICE OR POLICY**

- These findings have important implications for the design and implementation of interventions for populations at risk of poor mental and sexual health, including during international crises.

## Introduction

 An outbreak of a novel virus, SARS-CoV-2, causing COVID-19, was declared a pandemic by the WHO on 11 March 2020.^1^ Initially, no vaccines or treatments existed, meaning non-pharmaceutical interventions (NPIs), including physical distancing, face-coverings and lockdowns, were implemented to reduce transmission. The UK government announced a national lockdown on 23 March 2020. Restrictions were gradually eased, with regional variation, from June 2020,^2^ although some NPIs remained in place into 2022. The direct effects of the virus, in addition to the social and economic consequences of lockdowns, have affected all areas of people’s lives, including a range of inter-related impacts on physical and mental health.^3^,^8^

Population studies have shown that mental illness and psychological distress increased during the initial stages of the pandemic.^8^,^12^ Additionally, access to mental health services was reduced,^13 14^ and, where available, remote services may have excluded vulnerable people from diagnosis, treatment and monitoring and potentially increased existing mental health inequalities.

Social COVID-19 restrictions also impacted sexual behaviours and lifestyles, with effects varying depending on individual circumstances.^15^ During the most restrictive periods, those in cohabiting relationships spent more time with each other than before, and those living separately were prohibited from intimate contact. Additionally, the inclination to have sex may have been altered by the pandemic and associated stresses.^16 17^ Sexual risk behaviours, including those that increase the risk of sexually transmitted infections and/or unplanned pregnancy, may also have been affected by the pandemic, with reduced opportunity for meeting new sexual partners and reduced condom access.^3 18^ As for other services, access to inperson sexual and reproductive health services and treatment was reduced in the early stages of the pandemic, with a shift toward online and remote services.^19^

Long before COVID-19, adverse sexual health outcomes have been linked to poor mental health in a complex and bidirectional relationship. For example, while sexual function is thought to affect mental health, it has also been suggested that those with poor mental health report lower sexual satisfaction.^20^,^25^ Individuals with poor mental health may also be less interested in meeting new partners or less able to maintain relationships. Additionally, antidepressants and other medications may have adverse effects on sexual function.^26^ Sexual identity has also been linked to mental health, with sexual minorities reporting higher risk for poor mental health outcomes and mental illness increasing the risk of harmful behaviours.^23 27 28^

The sexual behaviours and sexual lifestyles associated with poor mental health during the early phase of the COVID-19 pandemic have been investigated among various populations. Contrasting associations between mental health and relationship status have been found.^29^,^31^ Sexual frequency,^17 32 33^ sexual identity,^3031 34^,^36^ sexual function^2933 37^,^40^ and sexual satisfaction^32 41 42^ were associated with poor mental health in various studies. Non-compliance with COVID-19 restrictions for intimate physical contact has also been linked to poor mental health.^43 44^ However, evidence is limited, with studies often using convenience sampling of specific groups, with non-validated measures of mental health outcomes, and the general population of adults in Britain has not been considered. This study, which measures both mental health and sexual health indicators in a large, quasirepresentative sample of adults living in Britain, fills this gap.

## Methods

### Study design

Natsal-COVID is a repeat, cross-sectional, quasi-representative web-panel survey of Britain’s general population aged 18–59 years, collecting information about sexual attitudes and lifestyles through the COVID-19 pandemic. The survey, designed by the National Survey of Sexual Attitudes and Lifestyles (Natsal) team, was run by research company Ipsos MORI and was carried out online. Questions were adapted from three previous decennial Natsal surveys and development work for Natsal-4. The full questionnaire is available at https://www.natsal.ac.uk/natsal-covid-study. Ethical approval was obtained from the University of Glasgow MVLS College Ethics Committee (ref: 20019174) and the London School of Hygiene and Tropical Medicine Research Ethics Committee (ref: 22565).

The target sample was 6500 people aged 18–59 years. Quotas for age group, gender, region and social grade were applied, aiming for a sample of 6000 participants, broadly representative of Britain’s population, as well as a 500-person boost in ages 18–29 years. The sample was weighted by age, gender, ethnicity, social grade and sexual identity, to help achieve a quasirepresentative sample. Full details of survey design and methods have been reported previously.^45^ Wave 1 data collection took place between 29 July 2020 and 10 August 2020 (4 months after the first lockdown began and after the introduction of bubbles/extended households for adults who lived alone or with no other adults in June 2020), with most questions concerning the period from the start of lockdown until the date of interview.

Poor mental health was assessed through the Generalised Anxiety Disorder 2 (GAD-2) questionnaire and Patient Health Questionnaire 2 (PHQ-2). These measures each ask two questions about symptoms of mental health disorders in the past 2 weeks, scored from 0 to 3, giving each a total possible score of 6. The GAD-2 and PHQ-2 perform well in screening for anxiety disorders (86% sensitivity and 83% specificity) and major depression (83% sensitivity and 92% specificity), respectively, with cut-off scores of ≥3.^46 47^ Anxiety and depression are the most common mental disorders and often co-occur.^48 49^ Combining GAD-2 and PHQ-2 scores results in one scale measuring psychological distress, the PHQ-4. This is efficient at detecting individuals who may be suffering from one or both of anxiety and depression, with scores ≥3, ≥6 and ≥9 representing mild, moderate and severe psychological distress, respectively.^49 50^

Six self-reported sexual behaviours and lifestyle factors were assessed for association with psychological distress—sexual frequency, sexual identity, sexual function, sexual satisfaction, sexual risk behaviours and intimate physical contact outside the household. Most questions about sexual behaviours asked about experiences since the start of lockdown (ie, the past 4 months). However, sexual frequency was defined as the reported number of occasions of sex (oral, vaginal or anal) in the previous 4 weeks. Sexual identity was measured using the Office for National Statistics harmonised standard.^51^ Sexual function was defined as how often participants experienced sexual difficulties (such as anxiety, pain, vaginal dryness, difficulty getting an erection/aroused, difficulty reaching climax or reaching climax too soon) since lockdown (never, not very often, sometimes, very often or always). Sexual satisfaction was defined as self-perceived changes to sex life satisfaction since lockdown (decreased a lot, decreased a little, stayed the same, increased a little or increased a lot). Sexual risk behaviours were the reported number of partners and reporting condomless sex with a new partner, since the start of lockdown. Intimate physical contact outside the household was defined as reported intimate physical contact (including kissing and oral/anal/vaginal sex or other genital contact) with a romantic or sexual partner outside the household in the past 4 weeks.

### Statistical analysis

All statistical analyses were carried out using the complex survey functions in Stata (V. 16.1) to account for weighting of the data. Summary descriptive statistics were calculated using the weighted samples and presented as percentages.

Prevalence of psychological distress was stratified by age, gender and relationship status. The associations between sexual behaviours and lifestyles and psychological distress were then evaluated using logistic regression. The analysis used a PHQ-4 binary threshold score of ≥9, indicating severe psychological distress, corresponding with the usual severity threshold for the GAD-2 and PHQ-2 measures when used independently (sensitivity analysis was also carried out comparing the different possible threshold scores). Crude ORs and age, gender and relationship status-adjusted ORs (AORs), as well as 95% CIs and global p values (using Wald tests), were calculated. These adjustments were decided a priori to assess which sexual behaviours and lifestyle factors were associated with poor mental health among British adults, during the early phase of the COVID-19 pandemic.

### Patient and public involvement

Neither patients nor the public were involved in the design, conduct, reporting or dissemination plans of our research, given the tight turnaround to complete the fieldwork in the context of the pandemic.

## Results

**Figure 1:**
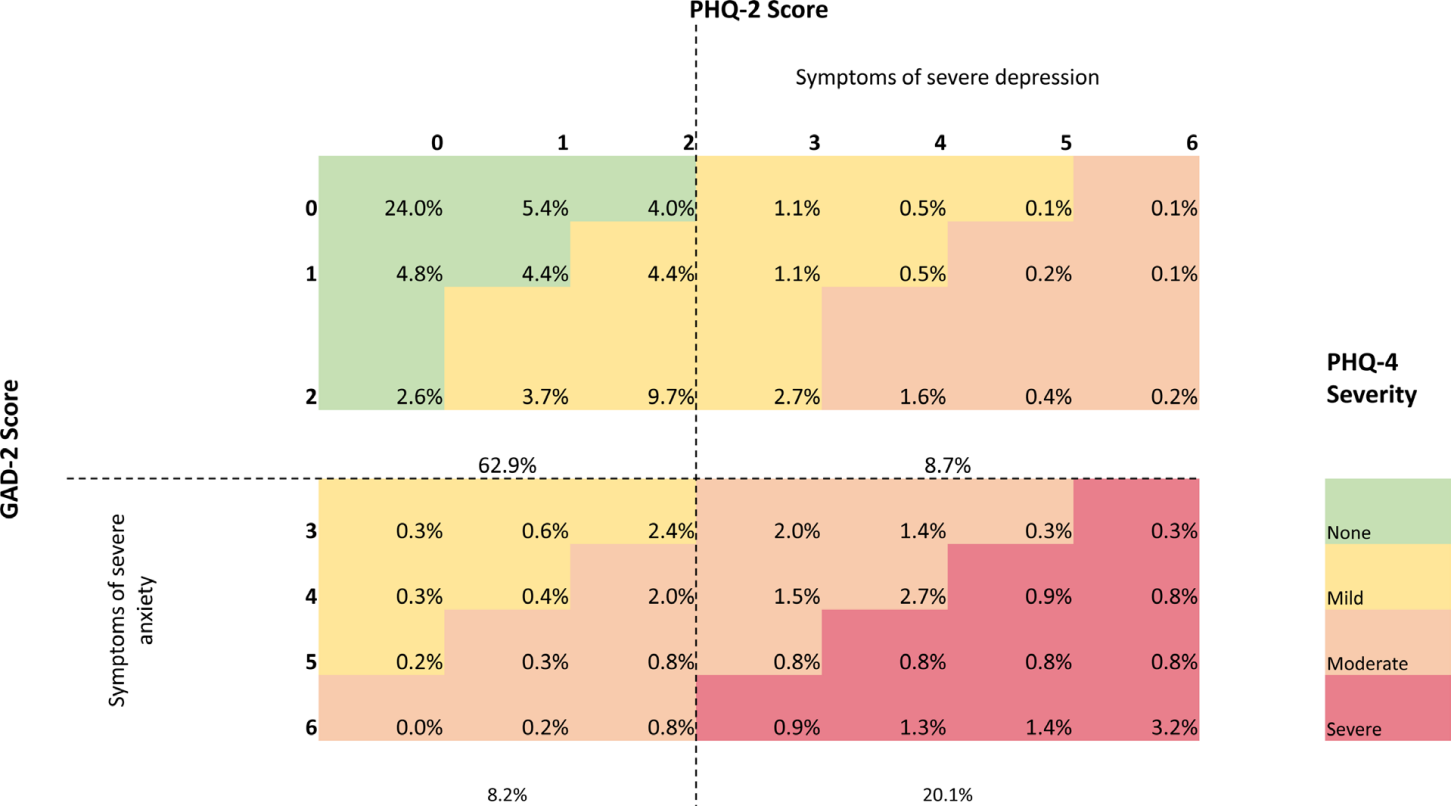
Weighted prevalence (%) of GAD-2 and PHQ-2 scores and the corresponding PHQ-4 severity score, among a quasirepresentative sample of British adults during the early phase of the COVID-19 pandemic. Of the 6654 Natsal-COVID participants, 6500 answered both the GAD-2 and PHQ-2, allowing the calculation of PHQ-4 scores which are represented in the figure. Dotted lines represent the GAD-2 and PHQ-2 threshold score (≥ 3) representing symptoms of severe anxiety and depression, respectively. The GAD-2 and PHQ-2 ask: over the following weeks, how often have you been bothered by the following problems? PHQ-2: little interest or pleasure in doing things, and feeling down, depressed or hopeless. GAD-2: feeling nervous, anxious or on edge and not being able to stop or control worrying. Possible answers and their corresponding scores: not at all, 0; several days, 1; more than half the days, 2; or nearly every day, 3. Scores for each measure are added for a total score, with a potential total of 6. GAD, Generalised Anxiety Disorder; PHQ, Patient Health Questionnaire.

Natsal-COVID had 6654 participants, whose characteristics are presented in table 1. Of these, 6543 completed the questions to derive PHQ-2 (29.1% reported symptoms of severe depression) and 6570 for GAD-2 (28.8% reported symptoms of severe anxiety). 6496 participants answered both the GAD-2 and PHQ-2, enabling the calculation of a PHQ-4 score, with more than half of the population (54.8%) reporting psychological distress (figure 1).

**Table 1:** Demographic and physical health characteristics of a quasirepresentative sample of 6654 British adults during the early phase of the COVID-19 pandemic

	Proportion of sample		Denominator	
	%	CI	Weighted	Unweighted
**Demographics**				
Age group (years)			6654	6654
18–24	12.9	(12.1, 13.8)	860	1008
25–29	15.0	(14.2, 15.9)	1000	1206
30–39	24.3	(23.2, 25.5)	1618	1508
40–49	23.0	(21.9, 24.1)	1530	1434
50–59	24.7	(23.6, 25.9)	1645	1498
Gender*			6654	6654
Men	49.7	(48.5, 51.0)	3310	3187
Women	49.9	(48.6, 51.2)	3320	3443
In another way	0.4	(0.2, 0.5)	24	24
Ethnicity			6528	6528
White†	85.7	(84.6, 86.7)	5593	5837
Mixed/multiple/other‡	2.8	(2.4, 3.4)	185	169
Asian / Asian British§	8.1	(7.3, 8.9)	530	395
Black / Black British¶	3.4	(2.8, 4.0)	221	127
Education			6654	6654
Degree	47.3	(46.0, 48.6)	3149	3191
Below degree	48.4	(47.1, 49.7)	3221	3195
No qualifications	4.3	(3.7, 4.8)	283	268
Employment			6654	6654
Employed	75.2	(74.1, 76.3)	5007	5000
Unemployed	14.1	(13.2, 15.0)	935	924
Full time parent or carer	5.7	(5.1, 6.3)	379	345
Student	5.0	(4.5, 5.6)	332	385
Rurality			5735	5741
Urban	85.4	(84.4, 86.3)	4896	4895
Rural	14.6	(13.7, 15.6)	840	846
Sexual Identity				
Heterosexual/straight	96.0	(95.6, 96.3)	6291	5762
Homosexual/gay/lesbian	1.8	(1.6, 2.0)	118	326
Bisexual	1.4	(1.3, 1.6)	93	393
Other	0.8	(0.6, 1.0)	51	74
**Physical health**				
General health			6640	6639
Good–very good	73.3	(72.2, 74.5)	4870	4846
Fair	21.1	(20.1, 22.2)	1400	1419
Bad–very bad	5.6	(5.0, 6.2)	370	374
Disability**			6538	6537
No disability	67.7	(66.5, 68.9)	4428	4310
Non-limiting disability	9.2	(8.5, 10.0)	603	620
Limiting disability	23.0	(22.0, 24.1)	1506	1607
COVID-19 experience (symptoms and/or diagnosis)			6271	6267
No	85.7	(84.8, 86.6)	5375	5319
Yes	14.3	(13.4, 15.2)	896	948
Alcohol Consumption (days drinking in last 7)			6654	6654
0	37.2	(35.9, 38.4)	2474	2407
1–2	36.3	(35.1, 37.6)	2417	2466
3–4	16.6	(15.7, 17.6)	1106	1118
5–7	9.9	(9.1, 10.7)	657	663

* Natsal-COVID was inclusive in its approach to gender and so data are presented separately for men (including trans men) and women (including trans women).

† White includes all those who identify as White English, Welsh, Scottish, Northern Irish, British, Irish, Gypsy or Irish Traveller or from any other white background.

‡ Mixed ethnicity includes those who identify as White and Black African, White and Black Caribbean, White and Asian or any other mixed or multiple ethnic background.

§ Asian includes those who identify as Indian, Pakistani, Bangladeshi, Chinese or from any other Asian background.

¶ Black includes those who identify as African, Caribbean or from any other Black background.

** Physical or mental health conditions lasting or expected to last for 12 months or more.

The prevalence and severity of psychological distress symptoms by age group, gender and relationship status is shown in table 2; overall, 45.2% of participants reported no symptoms, 28.0% mild psychological distress symptoms, 15.6% moderate and 11.3% reported severe symptoms. Participants who were younger, women and those not married/steady (living together) were more likely to report symptoms.

**Table 2:** Weighted prevalence of psychological distress, among a quasi-representative sample of British adults during the early phase of the COVID-19 pandemic

Psychological distress (PHQ-4)	None	Mild	Moderate	Severe	Denominator*
	% (95% CI)	% (95% CI)	% (95% CI)	% (95% CI)	Weighted, unweighted
Total	45.2% (43.9%,46.5%)	28.0% (26.8%,29.1%)	15.6% (14.6%,16.5%)	11.3% (10.5%,12.1%)	6496, 6500
Age group (years)					
18–24	32.4% (29.2%,35.9%)	31.1% (27.8%,34.5%)	22.8% (19.9%,25.9%)	13.8% (11.6%,16.3%)	814, 960
25–29	35.9% (32.9%,39.0%)	31.8% (29.0%,34.8%)	19.6% (17.2%,22.3%)	12.7% (10.7%,14.9%)	967, 1170
30–39	40.6% (37.9%,43.3%)	30.3% (27.9%,32.8%)	17.6% (15.6%,19.7%)	11.6% (10.0%,13.4%)	1586, 1479
40–49	51.0% (48.3%,53.7%)	25.4% (23.1%,27.8%)	12.5% (10.8%,14.5%)	11.1% (9.5%,12.9%)	1500, 1407
50–59	56.2% (53.6%,58.8%)	24.2% (22.0%,26.6%)	10.4% (8.9%,12.1%)	9.2% (7.8%,10.8%)	1629, 1484
Gender†					
Men	48.8% (46.9%,50.7%)	24.8% (23.2%,26.4%)	15.9% (14.6%,17.4%)	10.5% (9.4%,11.7%)	3214, 3100
Women	41.7% (39.9%,43.5%)	31.2% (29.5%,32.9%)	15.2% (13.9%,16.5%)	11.9% (10.8%,13.1%)	3257, 3376
Relationship status					
Married/steady (living together)	48.5% (46.8%,50.2%)	28.1% (26.6%,29.7%)	14.9% (13.8%,16.2%)	8.5% (7.6%,9.4%)	3808, 3751
Married/steady (not living together)	37.1% (32.7%,41.8%)	32.7% (28.5%,37.2%)	15.7% (12.6%,19.4%)	14.5% (11.4%,18.2%)	468, 510
Other (casual, new, >1, ending, other)	33.9% (28.5%,39.6%)	29.5% (24.3%,35.2%)	19.0% (14.8%,24.0%)	17.7% (13.6%,22.7%)	310, 331
Single	42.4% (40.0%,44.9%)	26.2% (24.1%,28.4%)	16.3% (14.5%,18.2%)	15.1% (13.5%,16.9%)	1895, 1896

* Of 6654 Natsal-COVID participants, 6500 answered both the GAD-2 and PHQ-2, enabling the calculation of a PHQ-4 score.

† Natsal-COVID was inclusive in its approach to gender and so data are presented separately for men (including trans men) and women (including trans women). We do not present separate estimates for participants who identified ‘in another way’ due to small numbers (24 participants).

GAD, Generalised Anxiety Disorder questionnaire; PHQ, Patient Health Questionnaire.

Several demographic factors were independently associated with severe psychological distress (table 3). Odds of severe psychological distress decreased with age group and were higher for those with below degree-level (AOR 1.38) and no qualifications (AOR 2.46) compared with those with degree-level education. The odds of severe psychological distress varied by employment status and were highest for unemployed participants (AOR 3.02). No significant association was found with gender or ethnicity, and association with rurality was non-significant after age-adjustment.

**Table 3:** Associations of demographic and health factors with severe psychological distress (measured using PHQ-4) among a quasirepresentative sample of British adults during the early phase of the COVID-19 pandemic

	% Sample	Severe psychological distress (%)	Crude OR (95% CI)	P value*	AOR† (95% CI)	P value*	Denominator‡ (weighted, unweighted)
**Demographics**							
Age group (years)				0.012			(6496, 6500)
18–24	12.9	13.3	1.57 (1.20 to 2.06)				(814, 960)
25–29	15.0	12.3	1.43 (1.10 to 1.86)				(967, 1170)
30–39	24.3	11.4	1.29 (1.01 to 1.63)				(1586, 1479)
40–49	23.0	10.9	1.23 (0.95 to 1.58)				(1500, 1407)
50–59	24.7	9.2	1.00				(1629, 1484)
Gender§				0.075		0.070	(6472, 6476)
Men	49.9	10.3	1 00		1.00		(3214, 3100)
Women	50.1	11.8	1.15 (0.98 to 1.36)		1.16 (0.99 to 1.37)		(3257, 3376)
Ethnicity				0.695		0.257	(6396, 6398)
White¶	85.7	11.3	1.00		1.00		(5489, 5731)
Mixed/multiple/other**	2.8	8.8	0.77 (0.42 to 1.39)		0.58 (0.31 to 1.12)		(180, 164)
Asian / Asian British††	8.1	10.1	0.9 (0.63 to 1.28)		0.79 (0.55 to 1.14)		(515, 382)
Black / Black British‡‡	3.4	12.7	1.17 (0.68 to 2.03)		0.93 (0.53 to 1.62)		(211, 121)
Education				<0.001		<0.001	(6496, 6500)
Degree	47.3	9.1	1.00		1.00		(3090, 3136)
Below degree	48.4	12.4	1.41 (1.19 to 1.68)		1.38 (1.16 to 1.64)		(3136, 3110)
No qualifications	4.3	19.4	2.43 (1.72 to 3.43)		2.46 (1.73 to 3.52)		(270, 254)
Employment				<0.001		<0.001	(6496, 6500)
Employed	75.2	8.9	1.00		1.00		(4900, 4900)
Unemployed	14.1	23.0	3.08 (2.54 to 3.74)		3.02 (2.46 to 3.71)		(909, 898)
Full time parent or carer	5.7	11.2	1.29 (0.89 to 1.88)		1.46 (0.99 to 2.15)		(369, 336)
Student	5.0	11.8	1.37 (0.96 to 1.95)		0.8 (0.55 to 1.18)		(318, 366)
Rurality				0.045		0.100	(5628, 5640)
Urban	85.4	11.2	1.00		1.00		(4799, 4805)
Rural	14.6	8.8	0.76 (0.58 to 0.98)		0.8 (0.61 to 1.04)		(829, 835)
**Health**							
General health				<0.001		<0.001	(6492, 6489)
Good–very good	73.3	6.8	1.00		1.00		(4743, 4764)
Fair	21.1	18.0	3.03 (2.52 to 3.66)		3.31 (2.73 to 4.01)		(1361, 1379)
Bad–very bad	5.6	41.7	9.79 (7.62 to 12.58)		11.46 (8.77 to 14.98)		(370, 365)
Disability§§				<0.001		<0.001	(6400, 6404)
No disability	67.7	6.3	1.00		1.00		(4342, 4228)
Non-limiting disability	9.2	7.8	1.26 (0.89 to 1.78)		1.37 (0.97 to 1.96)		(593, 609)
Limiting disability	23.0	25.8	5.25 (4.39 to 6.27)		5.61 (4.67 to 6.73)		(1465, 1567)
COVID-19 Experience (symptoms and/or diagnosis)				<0.001			(6482, 6486)
No	85.7	10.2	1.00		1.00		(5365, 5253)
Yes	14.3	15.1	1.61 (1.33 to 1.96)		1.64 (1.35 to 2.00)		(1175, 1233)
Alcohol consumption (days drinking in last 7)				0.001		0.010	(6481, 6488)
0	37.2	12.9	1.00		1.00		(2291, 2328)
1–2	36.3	10.1	0.76 (0.63 to 0.92)		0.82 (0.67 to 0.99)		(2368, 2417)
3–4	16.6	8.7	0.65 (0.50 to 0.84)		0.74 (0.57 to 0.96)		(1075, 1090)
5–7	9.9	12.7	0.98 (0.74 to 1.29)		1.16 (0.87 to 1.54)		(647, 653)

Crude and age, gender and relationship status are presented.

* Wald test.

† Age, gender and relationship adjusted OR, except for gender which is only age adjusted.

‡ Of 6654 Natsal-COVID participants, 6500 answered both the GAD-2 and PHQ-2, enabling the calculation of a PHQ-4 score.

§ Natsal-COVID was inclusive in its approach to gender and so data are presented separately for men (including trans men) and women (including trans women). We do not present estimates for participants who identified ‘in another way’ due to small numbers (24 participants).

¶ White English, Welsh, Scottish, Northern Irish, British, Irish, Gypsy or Irish Traveller or from any other white background.

** White and Black African, White and Black Caribbean, White and Asian or any other mixed or multiple ethnic background.

†† Indian, Pakistani, Bangladeshi, Chinese or from any other Asian background.

‡‡ African, Caribbean or from any other Black background.

§§ Physical or mental health conditions lasting or expected to last for 12 months or more.

GAD, Generalised Anxiety Disorder questionnaire; PHQ, Patient Health Questionnaire.

Health factors were strongly associated with severe psychological distress (table 3). Compared with those who reported good or very good general health, odds of severe psychological distress were higher for those who reported fair (AOR 3.31) and bad or very bad (AOR 11.46) general health. Similarly, those who reported a limiting disability had much higher odds of severe psychological distress than those without a disability (AOR 5.61). Reporting COVID-19 experience (symptoms or diagnosis) was also associated with severe psychological distress (AOR 1.64). Compared with those who did not drink in the last 7 days, the odds of severe psychological distress were somewhat higher in those who drank alcohol 5–7 days (AOR 1.16), but significantly lower among those who reported drinking 1–2 days (AOR 0.82) or 3–4 days (AOR 0.74).

Associations between relationship status, sexual behaviours and lifestyles and poor mental health are shown in table 4. Compared with those who were married or in steady relationships and living together, all other groups had higher odds of severe psychological distress, including those who were married or in steady relationships and not living together (AOR 1.78), single (AOR 1.88) and in other types of relationship such as casual, new, ending or more than one relationship (AOR 2.22). No significant association was found between living with child family members during lockdown and psychological distress.

**Table 4:** Associations of relationships and sexual behaviours and lifestyles with severe psychological distress (measured using PHQ-4) among a quasirepresentative sample of British adults during the early phase of the COVID-19 pandemic

	% Sample	Severe psychological distress (%)	Crude OR (95% CI)	P value*	AOR† (95% CI)	P value*	Denominator‡ (weighted, unweighted)
**Relationships**							
Relationship status				<0.001		<0.001	(6481, 6488)
Married/steady (living together)	58.6	8.3	1		1		(3808, 3751)
Married/steady (not living together)	7.2	14.3	1.83 (1.36 to 2.47)		1.78 (1.31 to 2.43)		(468, 510)
Other (casual, new, >1, ending, other)	4.8	17.3	2.32 (1.66 to 3.25)		2.22 (1.58 to 3.12)		(310, 331)
Single	29.4	14.9	1.92 (1.61 to 2.30)		1.88 (1.56 to 2.25)		(1895, 1896)
Living with child family member(s) since lockdown				0.935		0.058	(6485, 6491)
No	70.5	11.1	1		1		(4569, 4693)
Yes	29.5	11.2	1.01 (0.84 to 1.21)		1.2 (0.99 to 1.46)		(1916, 1798)
**Sexual behaviours, demographics and lifestyles**							
Sexual frequency (occasions of sex in past 4 weeks)				<0.001		0.034	(5349, 5407)
0	48.6	13.7	1		1		(2596, 2556)
1	11.1	9.8	0.69 (0.51 to 0.94)		0.87 (0.62 to 1.21)		(591, 604)
2–4	19	9.1	0.63 (0.49 to 0.81)		0.8 (0.60 to 1.07)		(1019, 1041)
5+	21.2	7.6	0.51 (0.40 to 0.66)		0.64 (0.48 to 0.86)		(1144, 1206)
Sexual identity				<0.001		<0.001	(6411, 6416)
Heterosexual/straight	96	10.6	1		1		(6155, 5643)
Homosexual/gay/lesbian	1.8	13.6	1.32 (0.90 to 1.94)		1.24 (0.84 to 1.82)		(116, 318)
Bisexual	1.4	21.4	2.32 (1.75 to 3.09)		2.13 (1.61 to 2.82)		(90, 383)
Other	0.8	34	4.38 (2.40 to 7.97)		3.2 (1.60 to 6.41)		(72, 51)
Sexual function (sexual difficulties since lockdown)				<0.001		<0.001	(3746, 3872)
Never	45.3	7.9	1		1		(1708, 1751)
Not very often	24.3	6.8	0.85 (0.61 to 1.18)		0.84 (0.60 to 1.17)		(914, 943)
Sometimes	21.5	12	1.63 (1.23 to 2.16)		1.67 (1.26 to 2.23)		(800, 840)
Very often–always	8.9	23.4	3.75 (2.71 to 5.19)		3.9 (2.78 to 5.47)		(323, 338)
Sexual Satisfaction (changes to sex life satisfaction since lockdown)				<0.001		<0.001	(5403, 5471)
Decreased a lot	8.5	23.7	3.63 (2.79 to 4.73)		3.2 (2.42 to 4.24)		(455, 460)
Decreased a little	14.5	12.9	1.71 (1.33 to 2.20)		1.62 (1.25 to 2.10)		(783, 832)
Stayed the same	63.8	8	1		1		(3458, 3426)
Increased a little	9.1	10.5	1.37 (0.99 to 1.89)		1.4 (1.01 to 1.95)		(486, 516)
Increased a lot	4.2	15.2	2.13 (1.40 to 3.25)		2.28 (1.48 to 3.52)		(221, 237)
Total sexual partners in lockdown				<0.001		0.001	(5453, 5516)
0	42.2	14.1	1.8 (1.50 to 2.16)		1.42 (1.10 to 1.82)		(2296, 2253)
1	54.7	8.4	1		1		(3001, 3069)
2+	3.1	17.1	2.43 (1.56 to 3.78)		2.12 (1.33 to 3.39)		(156, 194)
Condomless sex (with at least one new partner since lockdown)				0.031		0.046	(4911, 4922)
No	97.8	11	1		1		(4808, 4808)
Yes	2.2	17.5	1.79 (1.05 to 3.05)		1.77 (1.01 to 3.12)		(103, 114)
Intimate physical contact outside the household (in past 4 weeks)				0.8		0.479	(6496, 6500)
No	90.5	11.1	1		1		(5878, 5799)
Yes	9.5	11.4	1.04 (0.80 to 1.36)		0.91 (0.69 to 1.19)		(617, 701)

Crude and age, gender and relationship status are presented.

* Wald test.

† Age, gender and relationship adjusted OR, except for relationship status which is only age adjusted.

‡ Of 6654 Natsal-COVID participants, 6500 answered both the GAD-2 and PHQ-2, enabling the calculation of a PHQ-4 score.

GAD, Generalised Anxiety Disorder questionnaire; PHQ, Patient Health Questionnaire.

Sexual behaviour, demographic and lifestyle factors were also associated with severe psychological distress. The odds of severe psychological distress decreased with increasing sexual frequency. The odds of severe psychological distress also varied by sexual identity—compared with those who identified as heterosexual, odds were higher for those who identified as bisexual (AOR 2.13) or other sexual identity (AOR 3.20), but odds were not significantly higher for those who identified as homosexual. This association did not differ by gender (data not shown). Increasing frequency of sexual difficulties was associated with increased odds of psychological distress (AORs for reporting difficulties ‘sometimes’: 1.67 and ‘always’: 3.90, both compared with ‘never’). When individuals of all other sexual identities were grouped together and compared with those who identified as heterosexual, their odds of psychological distress were higher (AOR 1.89). Compared with those who reported their sexual satisfaction had stayed the same, odds of psychological distress were highest for those who reported it decreased a lot (AOR 3.20), while also higher for those who reported it increased a lot (AOR 2.28), decreased a little (AOR 1.62) and increased a little (AOR 1.40).

Number of partners since lockdown was associated with severe psychological distress, but the pattern was not linear, being more common among those reporting either no (AOR 1.42) or two or more partners (AOR 2.12, compared with those reporting one partner). Reporting condomless sex with a new partner in lockdown was also associated with psychological distress (AOR 1.77, compared with those reporting not having condomless sex with a new partner). No association was found between reporting intimate physical contact outside the household and psychological distress.

In a model adjusted for age, gender, relationship status, sexual frequency, sexual identity, sexual satisfaction, sexual function and total partner numbers, higher odds for severe psychological distress were still observed among bisexual participants (2.07 (1.41–3.03)) compared with heterosexual participants, those reporting sometimes (1.61 (0.19–2.18)) or very often (3.28 (2.26–4.75)) having sexual difficulties compared with those never experiencing sexual difficulties and those whose sexual satisfaction decreased a little (1.49 (1.07–2.08)) or a lot (2.63 (1.77–3.89)) compared with those whose stayed the same.

Results from a sensitivity analysis, comparing the different threshold scores of psychological distress, showed similar associations (online supplemental appendix 1). We also did a sensitivity analysis investigating associations with PHQ-2 and GAD-2 as independent outcomes and found broadly similar patterns (online supplemental appendix 2). Similarly, no difference in associations was found between sexual risk behaviours and poor mental health when sensitivity analysis was run with only those who reported sex since lockdown as the denominator (AOR 1.99, 1.20–3.29) for two or more partners compared with one partner since lockdown (AOR 1.63, 0.83–3.19).

## Discussion

This study shows that more than half of adults in Britain reported psychological distress during the early stages of the COVID-19 pandemic, and 1 in 10 had severe symptoms. Lockdowns and physical distancing had various impacts on sexual behaviours and lifestyles, increasing the time that cohabiting partners spent together, while preventing non-cohabiting partners from meeting at all. We observed that sexual behaviours and lifestyles during the early stages of the COVID-19 pandemic were strongly associated with poor mental health. Higher sexual frequency was associated with reduced psychological distress, in agreement with the literature, although no studies adjusted for cohabitation with a partner, and the directionality of this association is not clear.^17 32 33 52^ Adjusting for relationship status attenuated the strength of the association, possibly because those in cohabiting relationships had more opportunities for sex during lockdown and were less likely to experience psychological distress. Odds of psychological distress varied by sexual identity, with higher levels in those who identified as bisexual or in another way, than in those who identified as heterosexual, although not significantly higher for those who identified as homosexual. However, when all other sexual identities were grouped together, they had higher odds of psychological distress than those identifying as heterosexual. This broadly agrees with previous research, which suggests that reduced access to supportive and safe communities and spaces, being forced to isolate with unaccepting and/or unsupportive families (resulting in conflict relating to sexual orientation) and lack of privacy to continue remote treatment for pre-existing mental health conditions might all contribute to mental distress.^30 31 34 35 53^ Sexual difficulties were associated with psychological distress, as in other studies.^2933 37^,^40 54^ This relationship may be bidirectional—sexual difficulties likely cause mental distress, while poor mental health and associated medications could affect sexual function.^25 26^ Greater changes to sexual satisfaction during lockdown were also associated with psychological distress, after adjusting for gender, cohabitation and relationship status. This contrasts with the literature (although evidence was sparse), where decreased but not increased sexual satisfaction was associated with worse mental health, although categorical rather than binary outcomes in the sex life satisfaction variable may account for this difference.^32 41 42^

Sexual risk behaviours were associated with poor mental health; reporting condom use with new partners and reporting one partner (rather than none or two or more) was protective, likely because of the lower risk of psychological distress associated with being in a steady relationship. No previous investigation into the associations between sexual risk behaviours and poor mental health during the COVID-19 pandemic was found for comparison; however, depression has been linked to increased sexual risk behaviours.^2355^,^57^ The association with condom use was moderately weak, which could be due to the relatively large proportion of participants reporting no partners since lockdown (44%), meaning condomless sex was also not reported during this time. Overall, around 1 in 10 participants reported intimate physical contact outside the household (this was not associated with psychological distress), which suggests that the vast majority of people, including nearly half of those in a steady non-cohabiting relationship, adhered to guidelines. Although previous research indicates an association between casual sex in contravention of COVID-19 restrictions and mental distress, effects of relationship status and cohabitation were not considered.^44 58^

Our prevalence estimates of depression, anxiety and psychological distress are in line with those reported by others during the COVID-19 pandemic.^8^,^1012^ The Adult Psychiatric Morbidity Survey 2014 found 15.7% of adults displayed symptoms of common mental disorders—around half the estimates from the early stages of the pandemic.^59^ This suggests an increase in mental disorders and psychological distress from prepandemic levels but could also reflect a population-level increase in poor mental health since 2014 or increased reporting for reasons such as reduced mental health stigma. Psychological distress was associated with many demographic factors, as well as relationship status and physical health. The odds of psychological distress were higher for participants who were younger, less educated, unemployed, reporting poor general health, disability and COVID-19 experience, as well as varying by relationship status. These findings broadly concur with other studies which identified being young, female or of low socioeconomic status, experiencing financial strain, living with young children, living in urban areas, SARS-CoV-2 infection and having a pre-existing chronic disease or mental illness as risk factors.^510^,^12 60^

This study fills a gap in the literature about the associations between sexual behaviours and lifestyles and poor mental health among adults in Britain during the early phase of the COVID-19 pandemic. The large quasirepresentative population sample, with quotas and weighting, was broadly representative of Britain’s population, increasing generalisability. However, this was a non-probability sample, so may not be representative of Britain’s population by non-quota factors, including mental health. Web panel surveys may not provide robust population prevalence estimates, especially for sensitive behaviours,^63^ even though associations are likely to be generalisable. Additionally, as the study was cross-sectional, causality cannot be inferred. Conducting the data collection online meant that those without internet access, computer illiterate or without sufficient privacy were excluded, potentially introducing selection bias. Additionally, the survey methodology meant that non-response bias cannot be assessed.^3^ Recall bias might have been introduced when considering extended time periods, and there is the possibility of recasting.

The mental health measures used were robust and validated.^46 47 49 50^ However, these are screening tools, not clinical diagnoses, and only assess symptoms over the past 2 weeks so may not reflect an individual’s wider pandemic experience. Previous mental health disorder diagnosis and treatment were not measured and might have confounded the associations. For example, individuals being treated for mental health disorders may have been more likely to experience sexual dysfunction as a side effect of treatment and also more likely to experience poor mental health during the COVID-19 pandemic.^26^ We also recognise that the COVID-19 pandemic situation has changed rapidly, and the associations observed might not have endured as restrictions or public perceptions changed.

The high prevalence of mental health disorders and psychological distress highlights the importance of mental health services and support during an international emergency such as the pandemic. Understanding the causes might inform planning of social interventions in the event of future COVID-19 waves, as well as for other international crises. Our study emphasises the strength of associations between sexual health and mental health, which might be important for planning interventions to support both. Identifying populations at risk of psychological distress might also help target support and treatment.

## Supplementary material

10.1136/bmjph-2024-001443 online supplemental file 1

## Data Availability

Data are available in a public, open access repository.
