# Supplementary material for: Sexual behaviours and lifestyles associated with poor mental health among British adults during the early phase of the COVID-19 pandemic: findings from a large, quasirepresentative, cross-sectional web-panel survey (Natsal-COVID)
Source: BMJ Public Health. 2025 May 12;3(1):e001443. doi: 10.1136/bmjph-2024-001443 (PMC12086888; doi:10.1136/bmjph-2024-001443)
Supplement: online supplemental file 1 [file bmjph-3-1-s001.docx]

# Appendices

Appendix 1: Sensitivity analysis of PHQ-4 threshold scores in the associations of demographics, physical health, relationships and sexual behaviours and lifestyles with psychological distress, after adjusting for age, gender and relationship status.

|  | **None vs Mild/Moderate/Severe** | | | **None/Mild vs Moderate/Severe** | | | **None/Mild/Moderate vs Severe** | | |
| --- | --- | --- | --- | --- | --- | --- | --- | --- | --- |
|  | **AOR**^†^ | **CI** | **P*** | **AOR**^†^ | **CI** | **P *** | **AOR**^†^ | **CI** | **P*** |
| **Demographics** |  |  |  |  |  |  |  |  |  |
| **Ethnicity** |  |  | 0.402 |  |  | 0.945 |  |  | 0.359 |
| White^±^ | 1.00 |  |  | 1.00 |  |  | 1.00 |  |  |
| Mixed/multiple/other^¶^ | 1.13 | [0.74-1.71] |  | 1.01 | [0.67-1.53] |  | 0.62 | [0.32-1.17] |  |
| Asian / Asian British^¥^ | 0.97 | [0.77-1.22] |  | 1.01 | [0.80-1.29] |  | 0.83 | [0.58-1.18] |  |
| Black / Black British^¤^ | 0.73 | [0.49-1.08] |  | 0.88 | [0.58-1.34] |  | 1.84 | [1.07-1.84] |  |
| **Education** |  |  | <0.001 |  |  | <0.001 |  |  | <0.001 |
| Degree | 1.00 |  |  | 1.00 |  |  | 1.00 |  |  |
| Below degree | 1.28 | [1.14-1.43] |  | 1.48 | [1.31-1.67] |  | 1.45 | [1.22-1.72] |  |
| No qualifications | 1.78 | [1.32-2.39] |  | 2.18 | [1.62-2.94] |  | 2.57 | [1.81-3.64] |  |
| **Employment** |  |  | <0.001 |  |  | <0.001 |  |  | <0.001 |
| Employed | 1.00 |  |  | 1.00 |  |  | 1.00 |  |  |
| Unemployed | 2.24 | [1.88-2.67] |  | 2.52 | [2.12-3.00] |  | 3.42 | [2.81-4.20] |  |
| Full Time Parent or Carer | 1.16 | [0.91-1.48] |  | 1.62 | [1.24-2.12] |  | 1.43 | [0.98-2.08] |  |
| Student | 0.62 | [0.48-0.82] |  | 0.75 | [0.56-1.00] |  | 0.98 | [0.67-1.42] |  |
| **Rurality** |  |  | 0.002 |  |  | 0.003 |  |  | 0.074 |
| Urban | 1.00 |  |  | 1.00 |  |  | 1.00 |  |  |
| Rural | 0.77 | [0.66-0.91] |  | 0.75 | [0.62-0.91] |  | 0.79 | [0.60-1.02] |  |
| **Relationships** |  |  |  |  |  |  |  |  |  |
| **Living with Child Family Since Lockdown** | |  | 0.108 |  |  | 0.213 |  |  | 0.992 |
| No | 1.00 |  |  | 1.00 |  |  | 1.00 |  |  |
| Yes | 0.90 | [0.80-1.02] |  | 1.09 | [0.95-1.25] |  | 1.00 | [0.83-1.20] |  |
| **Physical Health** |  |  |  |  |  |  |  |  |  |
| **General Health** |  |  | <0.001 |  |  | <0.001 |  |  | <0.001 |
| Good-very good | 0.12 | [0.09-0.17] |  | 0.35 | [0.27-0.46] |  | 0.08 | [0.06-0.10] |  |
| Fair | 0.39 | [0.28-0.54] |  | 0.14 | [0.11-0.17] |  | 0.28 | [0.21-0.36] |  |
| Bad-very bad | 1.00 |  |  | 1.00 |  |  | 1.00 |  |  |
| **Disability** |  |  | <0.001 |  |  | <0.001 |  |  | <0.001 |
| No disability | 1.00 |  |  | 1.00 |  |  | 1.00 |  |  |
| Non-limiting disability | 1.69 | [1.39-2.05] |  | 1.43 | [1.15-1.80] |  | 1.39 | [0.98-1.97] |  |
| Limiting disability | 5.32 | [4.55-6.22] |  | 4.63 | [4.02-5.35] |  | 5.81 | [4.85-6.96] |  |
| **COVID-19 (symptoms and/or diagnosis)** | |  | <0.001 |  |  | <0.001 |  |  | <0.001 |
| No | 1.00 |  |  | 1.00 |  |  | 1.00 |  |  |
| Yes | 1.84 | [1.59-2.13] |  | 1.72 | [1.49-1.99] |  | 1.57 | [1.29-1.90] |  |
| **Alcohol Consumption (days drinking in last 7)** | |  | 0.689 |  |  | 0.187 |  |  | <0.001 |
| 0 | 1.00 |  |  | 1.00 |  |  | 1.00 |  |  |
| 1-2 | 0.99 | [0.87-1.13] |  | 0.99 | [0.86-1.15] |  | 0.75 | [0.62-0.91] |  |
| 3-4 | 0.96 | [0.82-1.13] |  | 0.99 | [0.83-1.19] |  | 0.66 | [0.51-0.85] |  |
| 5-7 | 1.09 | [0.90-1.33] |  | 1.24 | [1.00-1.54] |  | 1.03 | [0.77-1.36] |  |
| **Sexual Behaviours and Lifestyles** |  |  |  |  |  |  |  |  |  |
| **Sexual Function (difficulties since lockdown)** |  |  | <0.001 |  |  | <0.001 |  |  | <0.001 |
| Never | 1.00 |  |  | 1.00 |  |  | 1.00 |  |  |
| Not very often | 1.79 | [1.50-2.13] |  | 1.23 | [1.00-1.53] |  | 0.82 | [0.59-1.14] |  |
| Sometimes | 2.78 | [2.29-3.38] |  | 2.27 | [1.85-2.79] |  | 1.60 | [1.21-2.12] |  |
| Very often-always | 5.66 | [4.10-7.81] |  | 5.14 | [3.91-6.76] |  | 3.54 | [2.55-4.93] |  |
| **Sexual Frequency (occasions of sex in past 4 weeks)** | | | <0.001 |  |  | 0.003 |  |  | <0.001 |
| 0 | 1.00 |  |  | 1.00 |  |  | 1.00 |  |  |
| 1 | 0.99 | [0.80-1.22] |  | 1.05 | [0.83-1.33] |  | 0.68 | [0.50-0.93] |  |
| 2-4 | 0.77 | [0.64-0.92] |  | 0.83 | [0.68-1.02] |  | 0.62 | [0.48-0.79] |  |
| 5+ | 0.73 | [0.61-0.87] |  | 0.72 | [0.59-0.88] |  | 0.49 | [0.38-0.63] |  |
| **Sexual Satisfaction (changes to sex life satisfaction since lockdown)** | | | <0.001 |  |  | <0.001 |  |  | <0.001 |
| Decreased a lot | 1.00 |  |  | 1.00 |  |  | 1.00 |  |  |
| Decreased a little | 0.93 | [0.70-1.23] |  | 0.75 | [0.58-0.98] |  | 0.46 | [0.33-0.63] |  |
| Stayed the same | 0.41 | [0.32-0.53] |  | 0.43 | [0.34-0.54] |  | 0.28 | [0.21-0.36] |  |
| Increased a little | 0.63 | [0.46-0.86] |  | 0.61 | [0.45-0.83] |  | 0.37 | [0.25-0.53] |  |
| Increased a lot | 0.63 | [0.43-0.92] |  | 0.92 | [0.64-1.34] |  | 0.56 | [0.35-0.90] |  |
| **Number of Partners in Lockdown** | |  | <0.001 |  |  | <0.001 |  |  | <0.001 |
| 0 | 1.00 |  |  | 1.00 |  |  | 1.00 |  |  |
| 1 | 0.83 | [0.71-0.97] |  | 0.84 | [0.70-1.00] |  | 0.54 | [0.45-0.65] |  |
| 2+ | 2.03 | [1.37-3.02] |  | 1.82 | [1.26-2.63] |  | 1.25 | [0.80-1.95] |  |
| **Condomless Sex with at least one new partner in Lockdown** | | | <0.001 |  |  | <0.001 |  |  | 0.075 |
| No | 1.00 |  |  | 1.00 |  |  | 1.00 |  |  |
| Yes | 3.78 | [2.15-6.62] |  | 2.34 | [1.48-3.70] |  | 1.63 | [0.95-2.79] |  |
| **Intimate Physical Contact Outside the Household** | | | 0.592 |  |  | 0.723 |  |  | 0.865 |
| No | 1.00 |  |  | 1.00 |  |  | 1.00 |  |  |
| Yes | 1.06 | [0.86-1.31] |  | 0.96 | [0.77-1.20] |  | 0.98 | [0.74-1.28] |  |
| **Sexual Identity** |  |  | <0.001 |  |  | <0.001 |  |  | <0.001 |
| Heterosexual / Straight | 1.00 |  |  | 1.00 |  |  | 1.00 |  |  |
| Homosexual / Gay / Lesbian | 1.21 | [0.92-1.59] |  | 1.05 | [0.80-1.40] |  | 1.29 | [0.87-1.90] |  |
| Bisexual | 2.32 | [1.75-3.07] |  | 2.09 | [1.66-2.65] |  | 2.20 | [1.67-2.00] |  |
| Other | 4.19 | [1.67-10.51] |  | 3.30 | [1.75-6.23] |  | 3.96 | [2.00-7.84] |  |
|  |  |  |  |  |  |  |  |  |  |
|  |  |  |  |  |  |  |  |  |  |
| * Wald test |  |  |  |  |  |  |  |  |  |
| † Odds ratio adjusted for age, gender and relationship status | | | |  |  |  |  |  |  |
| ± White includes all those who identify as White English, Welsh, Scottish, Northern Irish, British, Irish, Gypsy or Irish Traveller, or from any other white background. | | | | | | | | | |
| ¶ Mixed ethnicity includes those who identify as White and Black African, White and Black Caribbean, White and Asian, or any other mixed or multiple ethnic background. | | | | | | | | | |
| ¥ Asian includes those who identify as Indian, Pakistani, Bangladeshi, Chinese or from any other Asian background. | | | | | | | | | |
| ¤ Black includes those who identify as African, Caribbean, or from any other Black background. | | | | | | | | | |

Appendix 2: Binary logistic regression evaluating the associations of demographics, physical health, relationships and sexual behaviours and lifestyles with both anxiety and depression. Crude, age-adjusted and age, gender and relationship status-adjusted odds ratios are presented.

| **Category** | **Anxiety**^∝^ | | | | | | | | | | **Depression**^×^ | | | | | | | | | | | |
| --- | --- | --- | --- | --- | --- | --- | --- | --- | --- | --- | --- | --- | --- | --- | --- | --- | --- | --- | --- | --- | --- | --- |
|  | **Crude OR [CI]** | **P*** | **aOR**^†^  [CI] | | | **P*** | **AOR**^‡^  [CI] | | | **P*** | **Crude OR**  **[CI]** | | | **P*** | **aOR**^†^  [CI] | | | **P*** | **AOR**^‡^  [CI] | | | **P*** |
| **Demographics** |  |  |  | | |  |  | | |  |  | | |  |  | | |  |  | | |  |
| **Age Group** |  |  |  | | |  |  | | |  |  | | |  |  | | |  |  | | |  |
| 50-59 | 1.00 |  |  | | |  |  | | |  | 1.00 | | |  |  | | |  |  | | |  |
| 40-49 | 1.24  [1.03-1.49] |  |  | | |  |  | | |  | 1.16  [0.97-1.38] | | |  |  | | |  |  | | |  |
| 30-39 | 1.74  [1.46-2.07] |  |  | | |  |  | | |  | 1.58  [1.33-1.88] | | |  |  | | |  |  | | |  |
| 25-29 | 2.10  [1.74-2.53] |  |  | | |  |  | | |  | 1.90  [1.58-2.29] | | |  |  | | |  |  | | |  |
| 18-24 | 2.67  [2.20-3.24] | <0.001 |  | | |  |  | | |  | 2.45  [2.02-2.97] | | | <0.001 |  | | |  |  | | |  |
| **Gender** |  |  |  | | |  |  | | |  |  | | |  |  | | |  |  | | |  |
| Male | 1.00 |  | 1.00 | | |  |  | | |  | 1.00 | | |  | 1.00 | | |  |  | | |  |
| Female | 1.12 [1.00-1.25] | 0.054 | 1.13 [1.01-1.27] | | | 0.043 |  | | |  | 0.87 [0.77-0.97] | | | 0.014 | 0.87 [0.78-0.98] | | | 0.020 |  | | |  |
| **Ethnicity** |  |  |  | | |  |  | | |  |  | | |  |  | | |  |  | | |  |
| White^±^ | 1.00 |  | 1.00 | | |  | 1.00 | | |  | 1.00 | | |  | 1.00 | | |  | 1.00 | | |  |
| Mixed/multiple/other^¶^ | 1.32 [0.91-1.93] |  | 1.08 [0.74-1.59] | | |  | 0.98 [0.67-1.45] | | |  | 1.23 [0.84-1.81] | | |  | 0.95 [0.63-1.42] | | |  | 0.91 [0.60-1.38] | | |  |
| Asian / Asian British^¥^ | 1.16 [0.92-1.47] |  | 0.97 [0.77-1.23] | | |  | 0.96 [0.76-1.22] | | |  | 1.32 [1.05-1.66] | | |  | 1.11 [0.88-1.41] | | |  | 1.09 [0.86-1.39] | | |  |
| Black / Black British^¤^ | 1.13 [0.76-1.70] | 0.271 | 0.94 [0.63-1.40] | | | 0.982 | 0.91 [0.61-1.35] | | | 0.956 | 1.27 [0.85-1.88] | | | 0.051 | 1.07 [0.72-1.59] | | | 0.794 | 1.01 [0.68-1.50] | | | 0.852 |
| **Education** |  |  |  | | |  |  | | |  |  | | |  |  | | |  |  | | |  |
| Degree | 1.00 |  | 1.00 | | |  | 1.00 | | |  | 1.00 | | |  | 1.00 | | |  | 1.00 | | |  |
| Below degree | 1.29 [1.14-1.45] |  | 1.39 [1.23-1.56] | | |  | 1.37 [1.21-1.54] | | |  | 1.45 [1.29-1.64] | | |  | 1.56 [1.38-1.75] | | |  | 1.49 [1.32-1.68] | | |  |
| No qualifications | 1.95 [1.47-2.57] | <0.001 | 2.16 [1.61-2.90] | | | <0.001 | 2.17 [1.62-2.91] | | | <0.001 | 2.39 [1.81-3.16] | | | <0.001 | 2.53 [1.88-3.41] | | | <0.001 | 2.46 [1.83-3.32] | | | <0.001 |
| **Employment** |  |  |  | | |  |  | | |  |  | | |  |  | | |  |  | | |  |
| Employed | 1.00 |  | 1.00 | | |  | 1.00 | | |  | 1.00 | | |  | 1.00 | | |  | 1.00 | | |  |
| Unemployed | 1.97 [1.69-2.31] |  | 2.4 [2.03-2.84] | | |  | 2.3 [1.94-2.73] | | |  | 2.17 [1.86-2.54] | | |  | 2.59 [2.19-3.06] | | |  | 2.38 [2.00-2.82] | | |  |
| Full Time Parent or Carer | 1.19 [0.92-1.53] |  | 1.42 [1.09-1.84] | | |  | 1.39 [1.06-1.82] | | |  | 1.08 [0.83-1.40] | | |  | 1.27 [0.98-1.65] | | |  | 1.45 [1.11-1.90] | | |  |
| Student | 1.40 [1.09-1.79] | <0.001 | 0.8 [0.61-1.05] | | | <0.001 | 0.74 [0.56-0.98] | | | <0.001 | 1.25 [0.97-1.60] | | | <0.001 | 0.74 [0.56-0.97] | | | <0.001 | 0.64 [0.49-0.85] | | | <0.001 |
| **Rurality** |  |  |  | | |  |  | | |  |  | | |  |  | | |  |  | | |  |
| Urban | 1.00 |  |  | | |  |  | | |  |  | | |  |  | | |  |  | | |  |
| Rural | 0.71 [0.60-0.86] | <0.001 | 0.77 [0.64-0.92] | | | 0.005 | 0.76 [0.64-0.92] | | | 0.004 | 0.64 [0.53-0.77] | | | <0.001 | 0.69 [0.57-0.83] | | | <0.001 | 0.71 [0.58-0.85] | | | <0.001 |
| **Relationships** |  |  |  | | |  |  | | |  |  | | |  |  | | |  |  | | |  |
| **Relationship Status** |  |  |  | | |  |  | | |  |  | | |  |  | | |  |  | | |  |
| Married/Steady (living together) | 1.00 |  | 1.00 | | |  |  | | |  | 1.00 | | |  | 1.00 | | |  |  | | |  |
| Married/Steady (not living together) | 1.47 [1.19-1.82] |  | 1.23 [0.99-1.54] | | |  |  | | |  | 1.60 [1.29-1.98] | | |  | 1.36 [1.09-1.70] | | |  |  | | |  |
| Other (Casual, New, >1, Ending, Other) | 1.77 [1.37-2.28] |  | 1.57 [1.21-2.04] | | |  |  | | |  | 2.01 [1.56-2.60] | | |  | 1.85 [1.43-2.41] | | |  |  | | |  |
| Single | 1.37 [1.21-1.56] | <0.001 | 1.24 [1.09-1.42] | | | <0.001 |  | | |  | 1.66 [1.46-1.89] | | | <0.001 | 1.54 [1.35-1.76] | | | <0.001 |  | | |  |
| **Living with Child Family Since Lockdown** |  |  |  | | |  |  | | |  |  | | |  |  | | |  |  | | |  |
| No | 1.00 |  | 1.00 | | |  | 1.00 | | |  | 1.00 | | |  | 1.00 | | |  | 1.00 | | |  |
| Yes | 0.99 [0.87-1.12] | 0.860 | 0.97 [0.86-1.10] | | | 0.663 | 1.04 [0.91-1.19] | | | 0.527 | 0.92 [0.81-1.04] | | | 0.178 | 0.89 [0.78-1.01] | | | 0.074 | 1.02 [0.89-1.17] | | | 0.800 |
| **Physical Health** |  |  |  | | |  |  | | |  |  | | |  |  | | |  |  | | |  |
| **General Health** |  |  |  | | |  |  | | |  |  | | |  |  | | |  |  | | |  |
| Good-very good | 1.00 |  | 1.00 | | |  | 1.00 | | |  | 1.00 | | |  | 1.00 | | |  | 1.00 | | |  |
| Fair | 2.13  [1.86-2.44] |  | 2.61  [2.26-3.01] | | |  | 2.58  [2.23-2.98] | | |  | 2.20  [1.92-2.52] | | |  | 2.65  [2.30-3.06] | | |  | 2.59  [2.24-2.99] | | |  |
| Bad-very bad | 4.62  [3.66-5.82] |  | 6.74  [5.27-8.63] | | | <0.001 | 6.58  [5.13-8.44] | | | <0.001 | 5.54  [4.37-7.02] | | | <0.001 | 7.73  [6.00-9.95] | | | <0.001 | 7.75  [5.63-9.33] | | | <0.001 |
| **Disability** |  |  |  | | |  |  | | |  |  | | |  |  | | |  |  | | |  |
| No disability | 1.00 |  | 1.00 | | |  | 1.00 | | |  | 1.00 | | |  | 1.00 | | |  | 1.00 | | |  |
| Non-limiting disability | 1.32 [1.07-1.63] |  | 1.55 [1.25-1.92] | | |  | 1.54 [1.24-1.91] | | |  | 1.07 [0.86-1.33] | | |  | 1.22 [0.98-1.51] | | |  | 1.24 [1.00-1.55] | | |  |
| Limiting disability | 3.80 [3.33-4.33] | <0.001 | 4.69 [4.07-5.40] | | | <0.001 | 4.62 [4.01-5.33] | | | <0.001 | 3.33 [2.92-3.79] | | | <0.001 | 3.96 [3.45-4.55] | | | <0.001 | 3.94 [3.43-4.53] | | | <0.001 |
| **COVID-19 (symptoms and/or diagnosis)** |  |  |  | | |  |  | | |  |  | | |  |  | | |  |  | | |  |
| No | 1.00 |  | 1.00 | | |  | 1.00 | | |  | 1.00 | | |  | 1.00 | | |  | 1.00 | | |  |
| Yes | 1.88 [1.64-2.16] | <0.001 | 1.76 [1.53-2.03] | | | <0.001 | 1.8 [1.56-2.08] | | | <0.001 | 1.71 [1.49-1.97] | | | <0.001 | 1.58 [1.37-1.82] | | | <0.001 | 1.62 [1.41-1.87] | | | <0.001 |
| **Alcohol Consumption (days drinking in last 7)** | |  |  | | |  |  | | |  |  | | |  |  | | |  |  | | |  |
| 0 | 1.00 |  | 1.00 | | |  | 1.00 | | |  | 1.00 | | |  | 1.00 | | |  | 1.00 | | |  |
| 1-2 | 0.97 [0.85-1.11] |  | 0.96 [0.84-1.10] | | |  | 0.99 [0.86-1.14] | | |  | 0.96 [0.84-1.10] | | |  | 0.94 [0.82-1.07] | | |  | 0.95 [0.83-1.09] | | |  |
| 3-4 | 0.93 [0.78-1.10] |  | 0.94 [0.79-1.11] | | |  | 0.98 [0.82-1.17] | | |  | 0.90 [0.76-1.07] | | |  | 0.89 [0.75-1.06] | | |  | 0.91 [0.76-1.08] | | |  |
| 5-7 | 1 [0.82-1.22] | 0.846 | 1.13 [0.92-1.39] | | | 0.385 | 1.19 [0.97-1.47] | | | 0.314 | 1.02 [0.84-1.25] | | | 0.618 | 1.14 [0.92-1.40] | | | 0.157 | 1.14 [0.92-1.41] | | | 0.223 |
| **Sexual Behaviours and Lifestyles** |  |  |  | | |  |  | | |  |  | | |  |  | | |  |  | | |  |
| **Sexual Function (difficulties)** |  |  |  | | |  |  | | |  |  | | |  |  | | |  |  | | |  |
| Never | 1.00 |  | 1.00 | | |  | 1.00 | | |  | 1.00 | | |  | 1.00 | | |  | 1.00 | | |  |
| Not very often | 1.44 [1.18-1.75] |  | 1.36 [1.11-1.66] | | |  | 1.39 [1.13-1.70] | | |  | 1.22 [1.00-1.50] | | |  | 1.14 [0.93-1.40] | | |  | 1.18 [0.96-1.45] | | |  |
| Sometimes | 2.16 [1.78-2.63] |  | 2.11 [1.73-2.57] | | |  | 2.17 [1.78-2.65] | | |  | 1.94 [1.59-2.37] | | |  | 1.89 [1.54-2.31] | | |  | 2 [1.63-2.45] | | |  |
| Very often-always | 4.70 [3.62-6.10] | <0.001 | 4.27 [3.27-5.58] | | | <0.001 | 4.48 [3.42-5.87] | | | <0.001 | 4.09 [3.15-5.31] | | | <0.001 | 3.69 [2.82-4.82] | | | <0.001 | 4.02 [3.07-5.26] | | | <0.001 |
| **Sexual Frequency (occasions of sex in past 4 weeks)** | |  |  | | |  |  | | |  |  | | |  |  | | |  |  | | |  |
| 0 | 1.00 |  | 1.00 | | |  | 1.00 | | |  | 1.00 | | |  | 1.00 | | |  | 1.00 | | |  |
| 1 | 1.01 [0.83-1.24] |  | 1 [0.81-1.22] | | |  | 1.02 [0.82-1.28] | | |  | 0.92 [0.75-1.13] | | |  | 0.9 [0.73-1.11] | | |  | 1.06 [0.85-1.33] | | |  |
| 2 | 0.88 [0.70-1.10] |  | 0.84 [0.67-1.06] | | |  | 0.85 [0.67-1.09] | | |  | 0.73 [0.57-0.92] | | |  | 0.7 [0.55-0.89] | | |  | 0.84 [0.65-1.09] | | |  |
| 3-4 | 0.66 [0.52-0.82] |  | 0.63 [0.50-0.79] | | |  | 0.65 [0.51-0.84] | | |  | 0.71 [0.57-0.88] | | |  | 0.69 [0.56-0.86] | | |  | 0.85 [0.67-1.09] | | |  |
| 5-9 | 0.64 [0.51-0.79] |  | 0.59 [0.48-0.74] | | |  | 0.6 [0.48-0.76] | | |  | 0.59 [0.48-0.74] | | |  | 0.56 [0.45-0.69] | | |  | 0.68 [0.53-0.86] | | |  |
| 10+ | 0.84 [0.68-1.05] | <0.001 | 0.73 [0.59-0.92] | | | <0.001 | 0.75 [0.58-0.96] | | | <0.001 | 0.75 [0.60-0.94] | | | <0.001 | 0.66 [0.53-0.83] | | | <0.001 | 0.8 [0.62-1.03] | | | 0.014 |
| **Sexual Satisfaction (changes to sex life satisfaction since lockdown)** | | | | | |  |  | | |  |  | | |  |  | | |  |  | | |  |
| Decreased a lot | 1.00 |  | 1.00 | | |  | 1.00 | | |  | 1.00 | | |  | 1.00 | | |  | 1.00 | | |  |
| Decreased a little | 0.73 [0.57-0.94] |  | 0.71 [0.54-0.92] | | |  | 0.74 [0.57-0.96] | | |  | 0.71 [0.56-0.92] | | |  | 0.68 [0.53-0.89] | | |  | 0.74 [0.56-0.96] | | |  |
| Stayed the same | 0.40 [0.32-0.50] |  | 0.42 [0.34-0.53] | | |  | 0.45 [0.35-0.56] | | |  | 0.38 [0.31-0.47] | | |  | 0.39 [0.32-0.49] | | |  | 0.44 [0.35-0.56] | | |  |
| Increased a little | 0.69 [0.52-0.91] |  | 0.63 [0.47-0.84] | | |  | 0.68 [0.50-0.91] | | |  | 0.63 [0.47-0.83] | | |  | 0.58 [0.43-0.77] | | |  | 0.67 [0.50-0.91] | | |  |
| Increased a lot | 0.95 [0.67-1.34] | <0.001 | 0.8 [0.56-1.15] | | | <0.001 | 0.89 [0.61-1.28] | | | <0.001 | 0.84 [0.59-1.19] | | | <0.001 | 0.72 [0.50-1.02] | | | <0.001 | 0.84 [0.58-1.21] | | | <0.001 |
| **Number of Partners in Lockdown** |  |  |  | | |  |  | | |  |  | | |  |  | | |  |  | | |  |
| 0 | 1.00 |  | 1.00 | | |  | 1.00 | | |  | 1.00 | | |  | 1.00 | | |  | 1.00 | | |  |
| 1 | 0.77 [0.68-0.88] |  | 0.74 [0.65-0.84] | | |  | 0.76 [0.64-0.91] | | |  | 0.68 [0.60-0.78] | | |  | 0.66 [0.58-0.75] | | |  | 0.81 [0.68-0.97] | | |  |
| 2+ | 2.48 [1.77-3.47] | <0.001 | 2.08 [1.48-2.92] | | | <0.001 | 2.15 [1.51-3.06] | | | <0.001 | 1.78 [1.26-2.52] | | | <0.001 | 1.5 [1.06-2.13] | | | <0.001 | 1.61 [1.13-2.31] | | | <0.001 |
| **Condomless Sex with at least one new partner in Lockdown** | | |  | | |  |  | | |  |  | | |  |  | | |  |  | | |  |
| No | 1.00 |  | 1.00 | | |  | 1.00 | | |  | 1.00 | | |  | 1.00 | | |  | 1.00 | | |  |
| Yes | 3.88 [2.53-5.97] | <0.001 | 3.04 [1.98-4.68] | | | <0.001 | 3.21 [2.05-5.02] | | | <0.001 | 2.86 [1.86-4.40] | | | <0.001 | 2.28 [1.48-3.53] | | | <0.001 | 2.25 [1.44-3.52] | | | <0.001 |
| **Intimate Physical Contact Outside the Household** | |  |  | | |  |  | | |  |  | | |  |  | | |  |  | | |  |
| No | 1.00 |  | 1.00 | | |  | 1.00 | | |  | 1.00 | | |  | 1.00 | | |  | 1.00 | | |  |
| Yes | 1.32 [1.10-1.58] | 0.003 | 1.15 [0.95-1.38] | | | 0.150 | 1.02 [0.82-1.27] | | | 0.845 | 1.33 [1.11-1.60] | | | 0.002 | 1.16 [0.96-1.40] | | | 0.113 | 0.96 [0.77-1.20] | | | 0.751 |
| **Sexual Identity** |  |  |  | | |  |  | | |  |  | | |  |  | | |  |  | | |  |
| Heterosexual / Straight | 1.00 |  | 1.00 | | |  | 1.00 | | |  | 1.00 | | |  | 1.00 | | |  | 1.00 | | |  |
| Homosexual / Gay / Lesbian | 1.17 [0.89-1.53] |  | 1.15 [0.87-1.51] | | |  | 1.13 [0.86-1.49] | | |  | 1.20 [0.92-1.58] | | |  | 1.2 [0.91-1.58] | | |  | 1.1 [0.83-1.45] | | |  |
| Bisexual | 3.01 [2.38-3.80] |  | 2.20 [1.75-2.77] | | |  | 2.12 [1.68-2.67] | | |  | 2.20 [1.74-2.79] | | |  | 1.9 [1.51-2.39] | | |  | 1.89 [1.50-2.39] | | |  |
| Other | 4.38 [2.50-7.65] | <0.001 | 3.98 [2.07-7.62] | | | <0.001 | 3.66 [1.91-7.04] | | | <0.001 | 4.46 [2.53-7.87] | | | <0.001 | 3.93 [2.07-7.48] | | | <0.001 | 3.5 [1.89-6.47] | | | <0.001 |
| * Wald test |  |  |  | | |  |  | | |  |  | | |  |  | | |  |  | | |  |
| † Adjusted for age |  |  |  | | |  |  | | |  |  | | |  |  | | |  |  | | |  |
| ‡ Adjusted for age, gender and relationship status | |  | |  |  | | |  |  | | |  |  | | |  |  | | |  |  | |
| ± White includes all those who identify as White English, Welsh, Scottish, Northern Irish, British, Irish, Gypsy or Irish Traveller, or from any other white background. | | | | | | | | | | | | | | | | | | | | | | |
| ¶ Mixed ethnicity includes those who identify as White and Black African, White and Black Caribbean, White and Asian, or any other mixed or multiple ethnic background. | | | | | | | | | | | | | | | | | | | | | | |
| ¥ Asian includes those who identify as Indian, Pakistani, Bangladeshi, Chinese or from any other Asian background. | | | | | | | | | | | | | | | | | | | | | | |
| ¤ Black includes those who identify as African, Caribbean, or from any other Black background.  ∝ Of 6,654 Natsal-COVID participants, 6,570 answered the GAD-2 questions.  × Of 6,654 Natsal-COVID partiticpants, 6,543 answered the PHQ-2 questions. | | | | | | | | | | | | | | | | | | | | | | |
|  |  |  |  | | |  |  | | |  |  | | |  |  | | |  |  | | |  |
